# Establishing an Alzheimer's Cohort for South Asian and MENA Populations: Insights from the First Six Months of the SAMENA Study

**DOI:** 10.1002/alz70857_101552

**Published:** 2025-12-25

**Authors:** Yun Cai, Rebecca West, Sharon Sanz Simon, Akankshi Oberoi, Michal S Beeri

**Affiliations:** ^1^ Herbert and Jacqueline Krieger Klein Alzheimer's Research Center at Rutgers Brain Health Institute, New Brunswick, NJ, USA; ^2^ Herbert and Jacqueline Krieger Klein Alzheimer's Research Center at Rutgers Brain Health Institute, New Brunswick, NJ, USA; ^3^ Herbert and Jacqueline Krieger Klein Alzheimer's Research Center, New Brunswick, NJ, USA

## Abstract

**Background:**

South Asian and Middle Eastern/North African (MENA) populations remain severely underrepresented in Alzheimer's disease and related dementias (ADRD) research, despite evidence of significant disparities in risk factors, protective factors, and biomarker profiles across different races and ethnicities. Our SAMENA Cohort targets the offspring of ADRD patients from these populations, aiming to provide comprehensive, longitudinal data on ADRD risk and protective factors to inform tailored prevention and treatment strategies.

**Method:**

We implemented several strategies to recruit from South Asian and MENA communities: (1) community outreach through culturally specific radio and TV programs and health camps; (2) social media advertisements targeting high‐concentration areas; (3) building partnerships with community leaders, health organizations, and long‐term care facilities linked to these communities; and (4) distributing recruitment materials through Rutgers University and Robert Wood Johnson Medical Group networks. We also recently partnered with recruitment companies to leverage digital advertising and medical record mining for identifying potential participants.

**Result:**

Over six months, approximately 300 individuals contacted us. The primary reason for ineligibility was race/ethnicity, as 90% of those who reached out were White of European descent. We further screened 23 individuals and enrolled 12 participants, with 3 still in the consenting process. Of the 12 enrolled participants, 8 are South Asian and 4 are MENA, with ages ranging from 45 to 64 years. Six participants are female, and most (*n* = 8) hold advanced degrees. Baseline cognitive function, measured by the Montreal Cognitive Assessment (MoCA), represents normal cognition (mean = 28.4, SD=1.5).

**Conclusion:**

Initial recruitment efforts highlighted significant challenges in reaching South Asian and MENA populations. Due to the limited evidence on culturally tailored recruitment strategies in ADRD research, we will continue exploring barriers and facilitators by gathering perspectives from multiple stakeholders, including healthcare professionals, community leaders, and high‐risk offspring, to refine our recruitment strategies. Another approach is to expand our target group to include individuals with other Alzheimer's and dementia risk factors, such as obesity, diabetes/pre‐diabetes, and hypertension. The SAMENA study addresses a critical gap in ADRD research for these populations, whose underrepresentation may lead to missed early intervention opportunities, misdiagnoses from culturally insensitive assessments, and ineffective treatments.

